# Seasonal and diurnal variations of Kelvin-Helmholtz Instability at terrestrial magnetopause

**DOI:** 10.1038/s41467-023-37485-x

**Published:** 2023-05-04

**Authors:** S. Kavosi, J. Raeder, J. R. Johnson, K. Nykyri, C. J. Farrugia

**Affiliations:** 1 Embry–Riddle Aeronautical University, Daytona Beach, FL USA; 2 University of New Hampshire, Institute for the Study of Earth, Oceans and Space, Durham, NH USA; 3 Andrews University, Berrien Springs, MI USA

**Keywords:** Magnetospheric physics, Magnetospheric physics

## Abstract

Kelvin-Helmholtz Instability is ubiquitous at Earth’s magnetopause and plays an important role in plasma entry into the magnetosphere during northward interplanetary magnetic fields. Here, using one solar cycle of data from NASA THEMIS (Time History of Events and Macro scale Interactions during Substorms) and MMS (Magnetospheric Multiscale) missions, we found that KHI occurrence rates show seasonal and diurnal variations with the rate being high near the equinoxes and low near the solstices. The instability depends directly on the Earth’s dipole tilt angle. The tilt toward or away from the Sun explains most of the seasonal and diurnal variations, while the tilt in the plane perpendicular to the Earth‐Sun line explains the difference between the equinoxes. The results reveal the critical role of dipole tilt in modulating KHI across the magnetopause as a function of time, highlighting the importance of Sun-Earth geometry for solar wind-magnetosphere interaction and for space weather.

## Abstract (web summary)

Kelvin-Helmholtz Instability (KHI) has been suggested as a significant source of geomagnetic activity during northward Interplanetary Magnetic Fields (IMF). Here, the authors show seasonal and diurnal variations of KHI at Earth’s magnetopause, highlighting the importance of Sun-earth geometry for space weather.

## Introduction

There has been considerable debate over the causes of seasonal variation of geomagnetic activity. The seasonal variation with equinoctial maxima is explained mainly by three principal hypotheses; the axial hypothesis, which depends on heliographic latitude^1^, the equinoctial hypothesis, which depends on the dipole tilt angle^2–6^; the Earth’s dipole tilt toward or away from the Sun, and the Russell–McPherron (RM) effect^7^, which depends on the angle of the Earth’s dipole in the plane perpendicular to the Earth–Sun line. These mechanisms work in fundamentally different ways. The equinoctial hypothesis works by modifying the magnetosphere’s response to the solar wind; it reduces the coupling efficiency of the magnetosphere near solstices^8–11^. The RM effect creates seasonal variation by modifying the orientations of the Interplanetary Magnetic Field (IMF) via coordinate transformation; It leads to an enhancement of the southward component of the magnetic field during southward IMF conditions, increasing the geomagnetic activity associated with substorms near the equinoxes when this effect is most pronounced. While there are extensive studies on the seasonal variation of geomagnetic activity under southward IMF based on the occurrence rates and intensities of the magnetic substorms^12–15^, the seasonal feature of geomagnetic activity under northward IMF is still poorly understood. During northward IMF, viscous interaction of the solar wind with the magnetosphere and the KHI has been suggested as a significant source of geomagnetic activity and associated transport of mass, momentum, and energy^16–18^. The onset condition for KHI at the boundary between two magnetohydrodynamic incompressible fluids in relative motion is^19^.

1 $$ [{{{{{\bf{k}}}}}} \cdot ({{{{{\bf{V}}}}}}_{{{{{\rm{I}}}}}} - {{{{{{\bf{V}}}}}}}_{{{{{\rm{M}}}}}})]^{2} \, > \, \frac{{{\rho }_I}+{{\rho }_M}}{4\pi {{\rho }_I}{{\rho }_M}}\left[\left({{{{{\bf{k}}}}}} \cdot {{{{{{\bf{B}}}}}}}_{{{{{{\rm{I}}}}}}}\right)^2\,+\left({{{{{\bf{k}}}}}} \cdot {{{{{\bf{B}}}}}}_{{{{{{\rm{M}}}}}}}\right)^{2}\right]$$

**B** and *ρ* stand for magnetic field and mass density, respectively. **V** is the bulk velocity. The indices I and M are for the Interplanetary (Magnetosheath values) and Magnetosphere sides, respectively. The instability is driven by velocity shear aligned with the **k** vector, while the magnetic field component aligned with the **k** vector acts to stabilize the instability across the Magnetopause (MP) boundary. The stabilizing terms due to magnetic tension are the interplanetary term (**k** ⋅ **B**_I_), and the magnetospheric term (**k** ⋅ **B**_M_). The first term states the role of IMF as processed by the bow shock, thus resulting in the magnetosheath magnetic tension on the KHI. The second term expresses the role of Earth’s magnetic dipole field, the magnetosphere magnetic tension, on KHI. The contribution of these magnetic tension terms is minimized when the orientation of the magnetospheric field is perpendicular to the shear flow direction and when the interplanetary field and magnetospheric field become more aligned.

**Fig. 1:**
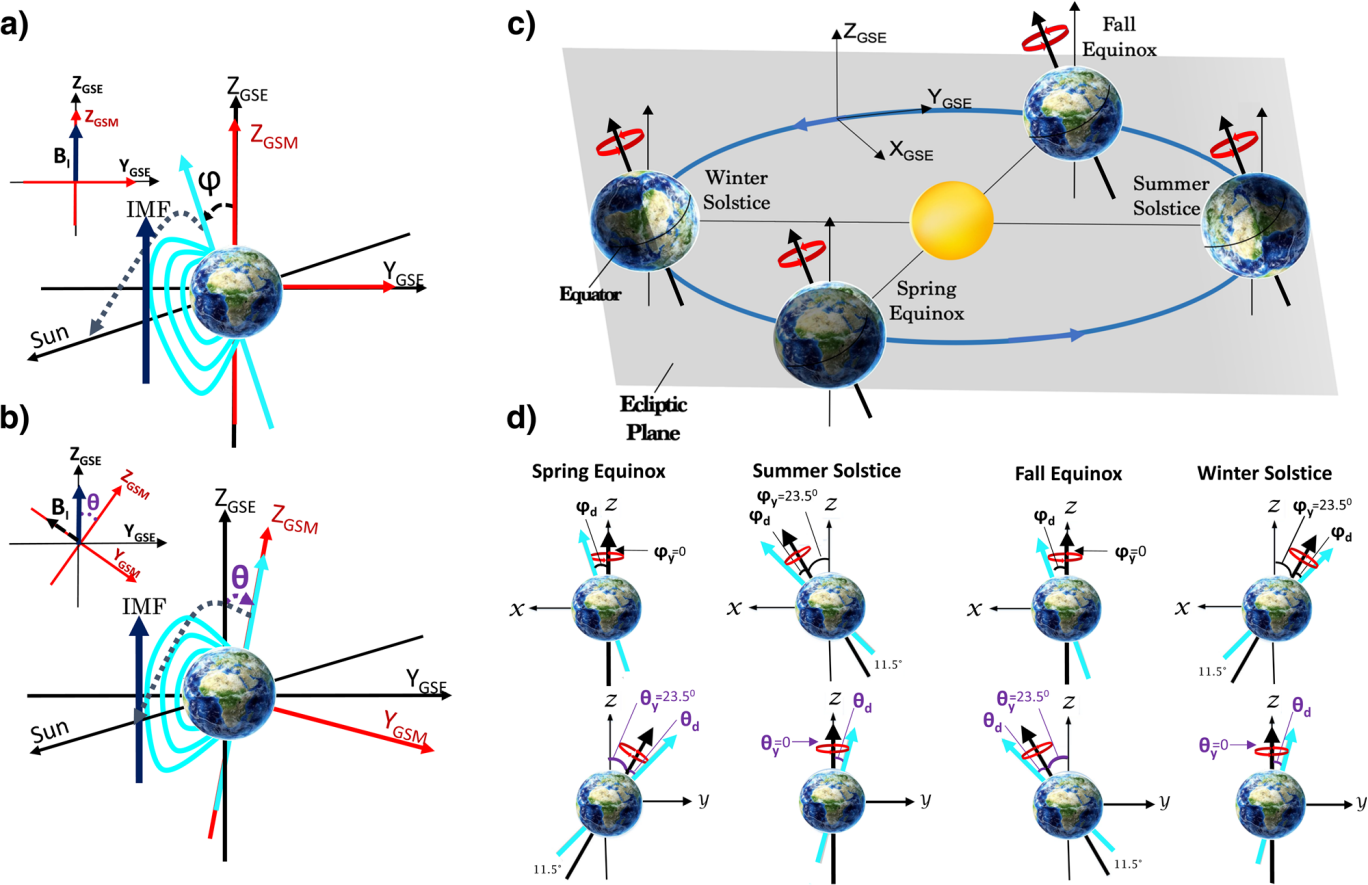
Schematic Sun–Earth geometry and seasonal and diurnal variations of the angles φ and θ. **a** Sketch of Earth’s magnetic dipole in both GSM and GSE coordinate systems at the summer solstice and **b** at the spring equinox. The angle that dipole axes make with the Sun–Earth line is shown with the black dashed line; it is (90° − φ) at the summer solstice shown in panel (**a**) and 90° at the spring equinox shown in panel (**b**). **c** Illustration of the Earth in its orbit around the Sun, showing the orientation of Earth’s rotational axis in black and defining the ecliptic plane (shaded gray) and the GSE reference frame. **d** The Earth’s rotational axis is shown as a black arrow, and the projection of the Earth’s magnetic axis is shown in blue. The rotational axis processes around Z_GSE_ once per year, and the magnetic axis precesses around the rotational axis every 24 h. The cone angles of these annual (θ_y_ and φ_y_) and daily (θ_d_ and φ_d_) precessions are 23.5 and 11.5°, respectively. The top panel shows the annual (φ_y_) and daily (φ_d_) variations of the angle φ in X-Z GSM for the spring equinox, summer solstice, fall equinox, and winter solstice. It shows views of Earth from dusk in the X-Z GSM plane. The bottom panel shows the annual (θ_y_) and daily (θ_d_) variations of angle θ in Y-Z GSE for the spring equinox, summer solstice, fall equinox, and winter solstice. It shows views toward Earth from the Sun in the Y-Z GSE plane.

Earth’s magnetic dipole axis is tilted toward or away from the Sun with a dipole tilt angle φ, the angle between the dipole axis and the Geocentric Solar Magnetospheric (GSM) Z-axis. The value of φ modulates the maximum and minimum of the magnetic tension term due to the magnetospheric field (**k** ⋅ **B**_M_). When the angle φ is 0°, the dipole is along the GSM Z-axis and perpendicular to the velocity shear (in the GSM X-Y plane), i.e., the magnetosphere magnetic tension is zero, and dipole field lines cannot exert a stabilizing influence on the KHI growth rate. These dipole orientations occur at the equinoxes on March 21 and September 21 at 10:30 and 22:30 UT, respectively. Figure 1a shows the most stable situation near the summer solstice at 16:30 UT when the dipole tilt φ is maximum (+35^°^). The parallel component of a magnetic dipole in the X-Z GSM plane is B_M_${{\sin }}$(φ) as illustrated in Fig. 1a.

The IMF term (**k** ⋅ **B**_I_) also contributes to the variation of the instability. This variation is explained in terms of the difference between the GSM and Geocentric Solar Ecliptic (GSE) systems. As the upstream IMF is rotated into GSM system, its orientation changes as a function of the angle of rotation, θ. The angle θ is the angle between the GSE and GSM Z-axis. The seasonal and diurnal variations of angle θ give rise to the variations of IMF orientations in the GSM coordinate system, leading to the variations of the magnetosheath magnetic tension term (**k** ⋅ **B**_I_), thus impacting the KHI growth rate. As the angle θ increases, the magnetosheath magnetic tension increases, exerting a stabilizing influence on KHI. Figure 1b displays the situation during the spring equinox on March 21 at 22:30 UT for purely northward IMF orientation. The parallel component of the IMF in the Y-Z GSM plane is B_I_sin(θ), as shown in Fig. 1b.

The Earth’s magnetic dipole orientation has both seasonal and diurnal variations due to the combined effects of the rotation of the Earth around the Sun and the rotation of the magnetic dipole about the Earth’s rotation axis. These effects are expected to introduce seasonal and diurnal variations for KHI growth by altering the intensity of the magnetic tension forces described above. Figure 1c illustrates Earth’s orbit at the equinoxes and solstices. In Fig. 1d, the top panel presents the seasonal (φ_y_) and diurnal (φ_d_) variations of the angle φ, and the bottom panel presents the seasonal (θ_y_) and diurnal (θ_d_) variations of the angle θ at the spring equinox, summer solstice, fall equinox, and winter solstice.

Here, we investigate the contribution of Sun–Earth geometry, the angles, θ, and φ on the occurrence rate of KHI at the Earth’s flank MP. For the first time, a comprehensive statistical analysis of 11 years (cycle 24) of in-situ data using THEMIS and MMS spacecraft during 2007–2018 is presented to address this objective. A comparison of the observational results with the theoretical analysis is performed to explain the mechanisms responsible for the seasonal and diurnal variations of KHI. We find that the KHI occurrence rate has seasonal dependences, with the maximum at the equinoxes and the minimum at the solstices, which are directly related to the dipole tilt angle φ. The difference in KHI occurrence rate between equinoxes and solstices arises from an equinoctial effect. In contrast, the difference in KHI occurrence rate between spring and fall Equinox arises from the angle θ between the GSM and GSE Z-axis.

## Results

### Seasonal variation of KHI occurrence

**Fig. 2:**
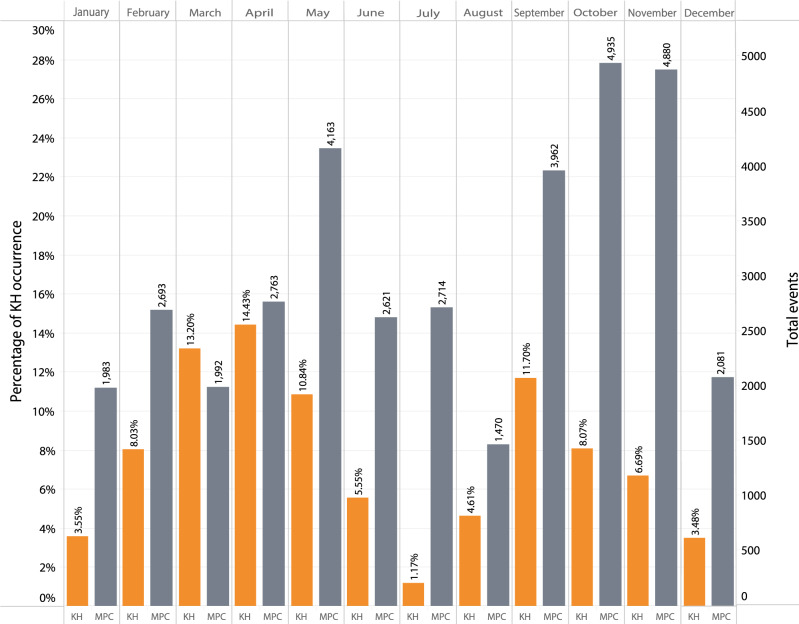
Seasonal variations of Kelvin–Helmholtz Instability at the flank magnetopause. Orange bins show the relative Kelvin–Helmholtz (KH) wave occurrence rate with respect to the number of boundary crossings, and the gray bins indicate the corresponding number of 5-min boundary crossing intervals in that bin. The rate maximizes at the equinoxes and minimizes at the solstices. Source data is provided as a Source Data File.

Figure 2 shows the percentage of KHI occurrence per month over the 11 years from 2007 to 2018, corresponding to solar cycle 24. The gray bars indicate the number of 5-min intervals in which the MP crossings (MPCs) were observed. The orange bars show the percentage of 5-min intervals where KH Waves (KHWs) are present. The gray bars are shown to assess the statistical significance of the data. The KHWs occurrence rate maximizes near equinoxes and minimizes near solstices. The highest occurrence rate is in March and April during the spring equinox season and in September and October during the fall season. The lowest occurrence rates occur during June, July, and August, i.e., near the summer solstice, and in January and December, i.e., near the winter solstice. The KHI occurrence rate near equinoxes is about three times greater than the solstices. This trend agrees well with the KHI growth rate predicted by theory. According to inequality (2), the most unstable configuration occurs at equinoxes; on March 21 at 10:30 and on September 21 at 22:30 UT, when φ is zero and θ is minimum (+11.5^°^ and −11.5^°^, respectively). The most stable configuration occurs near the winter solstices at 04:30 UT and summer solstices at 16:30 UT when the dipole tilt φ is maximum (−35^°^ and +35^°^, respectively).

### Diurnal variation of KHI occurrence

**Fig. 3:**
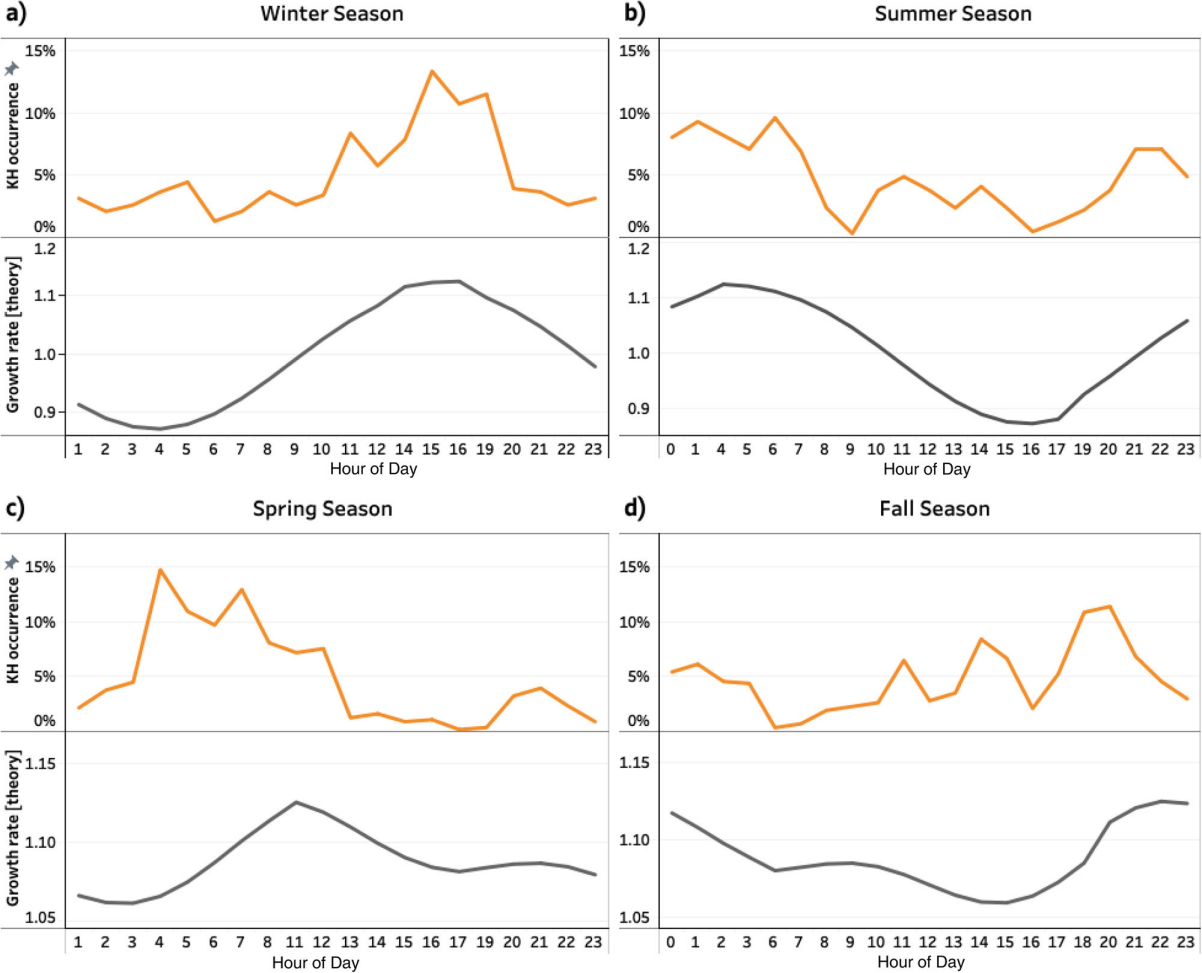
Diurnal variations of Kelvin–Helmholtz (KH) wave occurrences; comparison between observations and theoretical prediction. **a** Near the winter solstice, **b** summer solstice, **c** spring equinox, and **d** fall equinox. The orange lines show the diurnal variations from the observational dataset, and the black lines show the predicted growth rate based on the theory for comparison. Source data are provided as a Source Data file.

The times of the day at which maximum and minimum growth rates for the instability occur are seasonally dependent. The plots of the predicted KHI growth rate as a fraction of UT for different months of a year are shown in Supplementary Fig. 1. The normalized KHI occurrence rates using observational data (depicted by the orange lines) are plotted as a function of UT in Fig. 3a–d. The diurnal variation of the predicted KHI growth rate (depicted by the black lines) is also plotted for comparison. It should be noted that the statistical results provide the variation of the KHI occurrence rate, whereas inequality (1) provides the predicted KHI growth rates. Thus, a quantitative comparison is not possible. However, it is reasonable to expect that a large growth rate correlates with a high KHI occurrence rate.

The summer season consists of May, June, and July. The winter season consists of the months of November, December, and January. The spring season comprises the months of February, March, and April, and the fall season contains August, September, and October. The diurnal variation of KHI during the winter season is plotted in Fig. 3a for both observational data (orange line) and theory; the predicted growth rate (black line). Figure 3a shows that the maximum occurrence of KHI during the winter season occurs around 16:00 UT. This result agrees with the diurnal variation of the predicted growth rate depicted by the black line in Fig. 3a; near the winter solstice, the most unstable situation occurs around 16:00 UT, when φ is minimum. Figure 3b shows that the KHI occurrence rate during the summer season maximizes around 05:00 UT. This result also agrees with a predicted diurnal variation of the KHI growth rate; the most unstable situation occurs near the summer solstice when φ minimizes, around 04:00 UT. The trends in Fig. 3a, b show that the diurnal variation of KHI occurrence near the solstices exhibits an opposite direction; the minimums near the winter solstice are the maximums near the summer. This is expected from theory since the dipole tilt angle φ is in opposite directions during the solstice seasons (winter φ is negative, and the summer φ is positive).

Figure 3c and d shows the KHI occurrence rate as a function of UT during the spring and fall seasons, respectively. Figure 3c shows that the KHI occurrence rate has a double peak around 07:00 UT, and 21:00 UT, with a skew towards 07:00 UT. This result is slightly different (by approximately three hours) from the predicted KHI growth rate depicted by the black line, which shows a double peak around 10:00 UT and 21:00 UT with the skew towards 10:00 UT. During the fall season, shown in Fig. 3d, there is a similar double-peaked distribution (10:00 UT, and 21:00 UT) with a skew towards 21:00 UT. The origin of this skew in the distribution appears to be related to the magnetic tension associated with the angle of the Earth’s dipole in the plane perpendicular to the Earth–Sun line, the angle θ, as discussed earlier. This magnetic tension is maximized around 21:00 UT near the spring equinox and around 10:00 UT near the fall equinox. This effect breaks the symmetry of the double-peaked distribution expected from the dominant sin^2^φ near the equinoxes. Theoretical predictions of the KHI growth rates (black lines) are generally consistent with the statistical survey of KHI occurrence rates (orange lines). Diurnal variation near solstices, when the angle φ is large, is significantly larger than the times near equinoxes when the angle φ is small.

Supplementary Figure 2 displays the diurnal variation of KHI occurrences for the first six months of the year, Jan–June, and the second half of the year, July–Dec. The KHI occurrences maximize in the morning during Jan–June, while the situation is reversed during the second half of the year, July–Dec. These can be attributed to the angle θ, which is negative for the first half of the year and positive for the second half. A similar plot is presented for the time between the equinoxes, March–Aug, and Sep–Feb. The KHI occurrences maximize in the morning during March–Aug, and in the evening during Sep–Feb when the angle φ is positive and negative, respectively. Supplementary Figure 3 presents a similar plot but for the predicted growth rate for comparison. Therefore, the effect of the Earth’s dipole rotation around the spin axis appears to significantly affect the growth of KHI.

### Time-of-year and universal time pattern of KHI occurrence

To further verify the mechanism responsible for the diurnal and seasonal variations of KHI, the universal time (UT) and time-of-year (Y) variations of KHI occurrence are plotted for the entire dataset and compared with the Y-UT profiles of the angles φ and θ. The procedure involves the comparison between the observational pattern and the shape of the Y-UT plot of the predicted KHI growth rate. KHI growth rate can be represented as inequality (2).

2 $$\,\left[\left(\frac{4\pi {{\rho }_I}{{\rho }_M} \, \left(V_{I}\,-V_{M}\right)^{2}}{{{\rho }_I}+{{\rho }_M}}\,\right)-(({B}_{I}\sin (\uptheta ))^{2}+({B}_{M}\sin \left({{\upvarphi }}\right))^{2})\right].$$

Given that the first term in inequality (2) is constant, i.e., velocity shear and mass density are not sensitive to seasons, the Y-UT variations of the stabilizing term, [−(*B*^2^_I_sin^2^(θ) + *B*^2^_M_sin^2^(φ))], determines the Y-UT trend of KH growth rate.

**Fig. 4:**
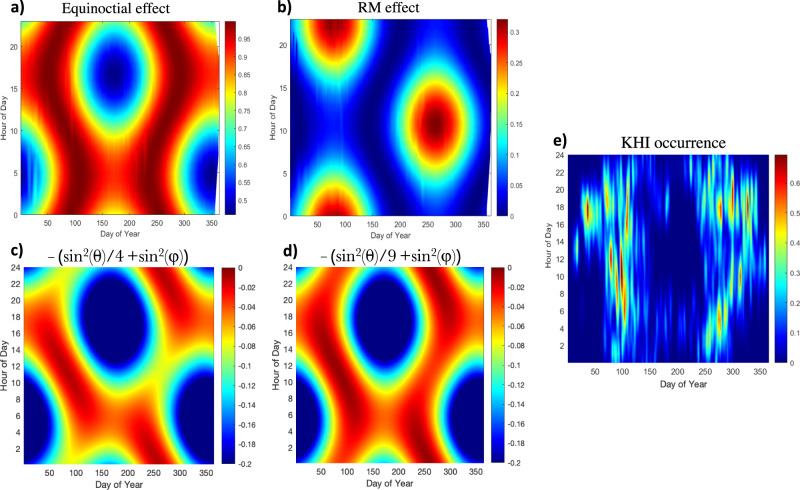
Time-of-year-time-of-day (Y-UT) plots. Of **a** cos^2^(φ), the equinoctial effect, **b** sin^2^(θ), the RM effect, [−(*B*^2^_I_sin^2^(θ) + *B*^2^_M_sin^2^(φ))] for **c** B_M_ = 2B_I_ and **d** B_M_ = 3B_I,_ respectively, with red showing when the stabilizing term is minimum, i.e., the growth rate is maximum. **e** KHI occurrence rates for one solar cycle data.

Figure 4a presents the Y-UT profiles of cos^2^(φ) and hence, the equinoctial pattern. Figure 4b illustrates the Y-UT profiles of sin^2^(θ), the classic RM pattern. Figures 4c and d show the Y-UT trend of the predicted growth rate, by plotting the Y-UT profile of the stabilizing term, [−(*B*^2^_I_sin^2^(θ) + *B*^2^_M_sin^2^(φ))], with red showing when the stabilizing term is minimum, i.e., the growth rate is maximum, for a different ratio of the magnetic tensions; B_M_ = 2*B*_I_ and B_M_ = 3*B*_I,_ respectively. The Y-UT plot of the KHI occurrence rate for the entire KHI dataset (solar cycle 24) is plotted in Fig. 4e for comparison to effectively identify the KHI seasonal and diurnal patterns. Figure 4e reveals an approximately similar overall shape compared to the equinoctial Y-UT pattern shown in Fig. 4a. Figure 4c, d shows how the equinoctial and RM effects are combined for a different ratio of the magnetic tension terms. It can be argued that the relative importance of the equinoctial effect vs. the RM effect depends on the ratio of the magnetic tensions (also proportional to B_M_ and B_I_). As B_M_/B_I_ becomes larger, as shown in Fig. 4e, the Y-UT profile becomes more comparable to the equinoctial effect. According to the observational (shown in Fig. 4e) and theoretical (shown in Fig. 4c, d) results, although the seasonal and diurnal variations of KHI may arise from the combined “Equinoctial/RM” effect, the equinoctial effect is the primary contributor and explains most of the seasonal variation.

## Discussion

It is shown that the observed seasonal and diurnal variations of KHI are caused by variations in Sun–Earth geometry; the angles φ and θ vary as Earth orbits around the Sun and rotates around its tilted axis. The semiannual and diurnal variations of these two angles play a vital role in KHI topology and occurrence. The equinoctial effect (based on an angle φ) works by modulating the magnetic dipole tension; it stabilizes KHI near solstices. Near the equinoxes, the tilt of the plane of the dipole into the ecliptic plane (angle θ) further modulates KHI occurrence by inducing the magnetic tension due to the magnetic field component of B_I_ in the GSM y-z plane. Since B_M_ is usually larger than B_I_ across the flank MP, the equinoctial effect has a more significant impact on the seasonal and diurnal variations of KHI, which is consistent with the Y-UT plot of KHI occurrence rate using one solar cycle KHI data, shown in Fig. 4e. It is also shown in Fig. 4c, d how the Y-UT profile of the predicted KHI growth rate changes as the ratio of the magnetic tensions due to φ and θ (also proportional to B_M_ and B_I_) changes, i.e., that it becomes more consistent with the equinoctial pattern. Moreover, it is generally expected that the **k** vector would mostly be confined to the shear flow plane as the **k** vector wants to align with the direction of the shear flow to maximize the KHI growth. The shear flow is largely in the X direction, thus the **k** vector. In that case, it can be argued that the magnetosphere magnetic tension (**k** ⋅ **B**_M_), due to the tilt of the dipole toward or away from the Sun, would be the dominant tension term and significantly more important for stabilizing the KHI. The tilt of the dipole into the ecliptic plane can become important when the dipole does not lean towards or away from the Sun near the equinoxes. The magnetic tensions maximize when the tilt is largest. As the magnetic tension in the plane perpendicular to the Earth–Sun line, a function of angle θ (similar to the RM effect) increases, the KHI growth rate decreases and vice versa. Consequently, the presence of the RM effect on KHI flattens the seasonal and diurnal variation curve by introducing an extra magnetic tension near equinoxes and no shear at solstices.

The seasonal variation of KHI, based on the theoretical analysis of inequality (1), was first proposed by Boller and Stolov^20^. Their analysis focused on the seasonal variation of the magnetospheric term (**k** ⋅ **B**_M_). The seasonal variation of the interplanetary term (**k** ⋅ **B**_I_) was not considered in their calculation because the seasonal variation of IMF had not been reported yet. We complemented the Boller and Stolov’s theoretical analysis by considering the seasonal variation of IMF (RM effect) in the analysis. The main assumption in the classic RM effect is that the southward IMF is the controlling factor of geomagnetic activity, and the northward IMF has no effect^7^. Therefore, although the angle θ contributes to the seasonal and diurnal variations of the KHI growth rate, the classic RM hypothesis fails to explain the variations of KHI, which frequently occur under northward IMF conditions. The dependence of seasonal and diurnal variations of KHI occurrences on the angle θ can be explained in terms of a change in IMF Clock Angle (CA) when it is rotated into the GSM coordinate system. IMF clock angle in GSM coordinate is a function of angle θ. KHI is more likely to occur at the MP when the IMF CA is small^21^. During northward IMF, the maximum change in CA(GSM) occurs near the equinoxes when θ maximizes. Thus, the variation of the IMF clock angle, when it is rotated into GSM coordinates, does explain how the KHI seasonal variations are related to the angle θ. In essence, the classic RM effect only predicts different probabilities of southward IMF at certain times of the year, while the mechanism proposed here predicts different probabilities of IMF CA at certain times of the year.

Note also that the seasonal dependence of λ, the heliographic latitude of the Earth, has not been considered in the present study. Several studies suggested a slight seasonal variation of solar wind velocity due to axial effect^22^. It was found that the solar wind velocity near Earth is higher at the northern and southern extremes of Earth’s heliographic latitude excursion (+−7.25^°^). If the seasonal variation of KHI is affected by the axial effect, it may be expected that the dates of maximum KHI occurrence will shift slightly from the equinoxes. However, much longer data sets will be required to examine the significance of the axial effect.

KHI transports solar wind plasma into the magnetosphere through rolled-up vortices^16^, drives magnetosphere Pc5 (2–7 mHz) Ultra Low Frequency (ULF) waves that can accelerate electrons to relativistic energies^23–25^, transports high-energy electrons and ions into the magnetosphere^26,27^, induce Field Aligned Current (FAC)^28^, and create auroral arcs^29^. In turn, the seasonal and diurnal variations in KHI occurrence will, in general, lead to temporal variation in the efficiency of solar wind-magnetospheric-ionosphere coupling during northward IMF conditions. As we shall see, this also affects the rest of the ionosphere-thermosphere system. These results will also be useful when designing spacecraft orbits and mission phases for future magnetospheric missions focusing on the KHI and secondary processes associated with the KHI that are responsible for plasma heating, acceleration, and transport^30,31^.

Earlier works based on in-situ observation^32^ reported that the magnetosphere preconditioning, the accumulation of cold-dense plasma sheet formation through processes such as KHI, leads to stronger-than-predicted storm activity during intervals of northward IMF. This, combined with the results presented in this study, may suggest that the more intense KHI activity around equinoxes leads to more plasma entry, which in turn may lead to more intense storms. Such a scenario would be consistent with the idea that the equinoctial and RM effect could operate simultaneously^33^ and that the RM effect is not the only explanation for the seasonal variation of geomagnetic activity^6,9^.

## Methods

### Statistical analysis

The study used measurements from THEMIS and MMS. The study is built on the previous database developed by Kavosi and Raeder^21^, the database of THEMIS KHI events from the beginning of the THEMIS mission to 2014. We expanded the database of MPCs/KHWs to one solar cycle (2007–2018) when the THEMIS spacecraft frequently crossed the MP during the dawn and dusk orbital phases. We also surveyed MMS from 2015 to 2018, when they were placed in a way that is in opposition with THEMIS. We inspected the magnetic field and plasma data to classify MPCs with the motivation to identify KHWs. Not all MP periodic oscillations are caused by KHI. Other mechanisms can excite periodic oscillations, such as dynamic pressure variations in the solar wind^34^ or Flux Transfer Events (FTEs)^35^. Thus, we needed to distinguish all MP wave observations against FTEs and buffeting of the magnetosphere by the solar wind. We examined solar wind data for every event, where possible, to confirm that the event was not caused by periodic solar wind pressure changes that may have caused buffeting. The KHW, in their nonlinear stage, has similar characteristics as FTEs, such as bipolar B_N_ and possibly similar wave periods of a few minutes. However, there are several differences in their signatures that allow us to distinguish rolled-up KH vortices from FTEs. When a KH wave grows to the nonlinear stage, it forms rolled-up vortices. In such vortices, the centrifugal force pushes plasma from the central part of the vortices radially out, generating a local minimum in the total pressure at the center of the vortex and maximum at the edge, thereby, substantial pressure perturbations, minimum at the vortex center and maximum at the edge is expected during KH vortices. In contrast, a total pressure maximum is expected at the FTE center. Although FTEs often occur in sequences, the individual FTEs are generally separated by a longer period of quiet. By contrast, KHWs are continuous wave trains. Note that this discussion only applies to non-linear KHW; in the linear stage, KHW can be easily discerned from FTEs by the lack of large bipolar B_N_ signatures and the absence of magnetic field magnitude and total pressure extrema. Scatter plots of the x (sunward) component of the velocity, V_X_, versus plasma density exhibits a distinct pattern, depending on the phase of the KHI growth. The scatter plot of V_X_ versus plasma density has also been performed for each event. We applied all methods discussed above to discriminate between FTEs and KHW, and whenever there was any ambiguity in the visual identification of an event, the event was categorized as MPCs. Data access and processing were done using SPEDAS V3.1, http://spedas.org/blog/.

Our KHWs database (Supplementary Data 1) consists of approximately 266 h of KHWs at MP (300 KHWs events), covering approximately 3200 5-min KHWs samples. The KHWs events last from 15 minutes (the shortest KHWs event in our database) to approximately 3 hours (the longest KHWs event in our database).

We have carefully assessed the seasonal locations of the THEMIS and MMS missions to identify and eliminate the possible orbital bias in the dataset. As shown in Supplementary Figs. 4 and 5, MMS spent more time near the dayside and tail during winter solstices and summer solstices, respectively. Such orbital bias may affect the statistics as KHWs expect to be more prevalent on the flanks than on the dayside. We defined a target region, −14 < x < +7Re, to avoid this orbital bias along the MP. The closest KHWs event to the subsolar point in our dataset is located at approximately +7 Re. The farthest KHWs from the dayside (KHWs near the tail flank) is located approximately at x = −14Re (see Supplementary Fig. 6). Therefore, any MPCs outside this region are excluded from the statistics to ensure that the smaller occurrence rate observed near solstices is not due to more MPCs recorded during those times.

### Theoretical analysis

Inequality (1) can be represented as:^20^

3 $$\left({{{{{V}}}}}_{{{{{{\rm{I}}}}}}}-{{{{{V}}}}}_{{{{{{\rm{M}}}}}}}\right)^{2} \, > \, \frac{{{\rho }_I}+{{\rho }_M}}{4\pi {{\rho }_I}{{\rho }_M}} \left[\left({{{{B}}}}_{{{{{\rm{I}}}}}}{\sin }\left({\uptheta }\right)\right)^{2}+({{{{{B}}}}}_{{{{{{\rm{M}}}}}}}{{\sin }}\left({{\upvarphi }}\right))^{2}\right]$$

assuming that the velocity shear is aligned with the **k** vector and is always in the GSM X-Y plane. Thus, the magnitude of inequality (2) determines the seasonal and diurnal variations of the KH growth rate. The inequality is computed in the regions where the value of velocity shear is sufficiently high; the regions around the subsolar point are excluded from the present discussion. The discussion focused on the parameters in inequality 2, which contribute to the seasonal and diurnal variations of the instability. Thus, the contributions of velocity, mass density, and IMF in the X direction (IMF cone angle) are excluded in the present discussion. We remind the reader that the GSM Y-axis is defined as perpendicular to the dipole axis so that the dipole axis is always contained in the X-Z plane. It is assumed for simplicity that the shear flow vector is parallel to the **k** vector. However, it has been reported that the **k** vector does not need to be aligned with the maximum shear flow but that KHWs can propagate with an angle from the shear flow to maximize the onset criteria^36,37^. A comprehensive analysis of the **k** vector orientation and its possible contributions to the seasonal and diurnal variations of KHI will be addressed in a later paper in this series. A detailed description of inequality 2 is presented in Supplementary Methods 1.

The magnitude of the stabilizing term, [−(*B*^2^_I_sin^2^(θ) + *B*^2^_M_sin^2^(φ))] has been solved numerically (Supplementary Code 1). The script presents the Y-UT trend of the predicted growth rate, [(constant) − (*B*^2^_I_sin^2^(θ) + *B*^2^_M_sin^2^(φ))], by plotting the variations of the stabilizing term, [−(*B*^2^_I_sin^2^(θ))^2^ + (*B*^2^_M_sin(φ))^2^], with red showing when the stabilizing term is minimum, i.e., the growth rate is maximum as shown in Fig. 4c, d. The magnitude of the stabilizing terms for different times of the day during the equinoxes and solstices are calculated and presented in Supplementary Table 1. The B_I_/B_M_ is assumed to be ½ for the theoretical prediction of diurnal variation shown in Fig. 3 (theory plot panels) and Supplementary Fig. 1. According to our observational dataset, during the time that KHWs are present at the MP, the average of the magnetosheath magnetic field magnitude at flank MP, B_I_, is approximately 20 nT, and the average magnitude of the magnetosphere magnetic field is about 50 nT. We used these numbers in our theoretical prediction.

## Supplementary information

Supplemetary Information

Description of Additional Supplementary Files

Supplementary Data 1

Supplementary Code 1

## Data Availability

The data that support the findings of this study are available from the corresponding author upon request. One solar cycle of the KHWs database from NASA THEMIS and MMS missions is provided in Supplementary Data 1. Source data are provided with this paper.

## Source data

Source Data
