# Supplementary material for: Seasonal and diurnal variations of Kelvin-Helmholtz Instability at terrestrial magnetopause
Source: Nat Commun. 2023 May 4;14:2513. doi: 10.1038/s41467-023-37485-x (PMC10160038; doi:10.1038/s41467-023-37485-x)
Supplement: Supplementary file 1 — Supplemetary Information [file 41467_2023_37485_MOESM1_ESM.pdf]

Supplementary Information on Seasonal and Diurnal variations of Kelvin-Helmholtz Instability at Terrestrial Magnetopause

S. Kavosi[1*], J. Raeder[2], Jay R. Johnson[3], Katariina Nykyri[1], C. J. Farrugia[2]

1 Embry–Riddle Aeronautical University, Daytona Beach, FL, USA
2 University of New Hampshire, Institute for the Study of Earth, Oceans and Space, Durham, NH, USA
3 Andrews University, Berrien Springs, MI, USA

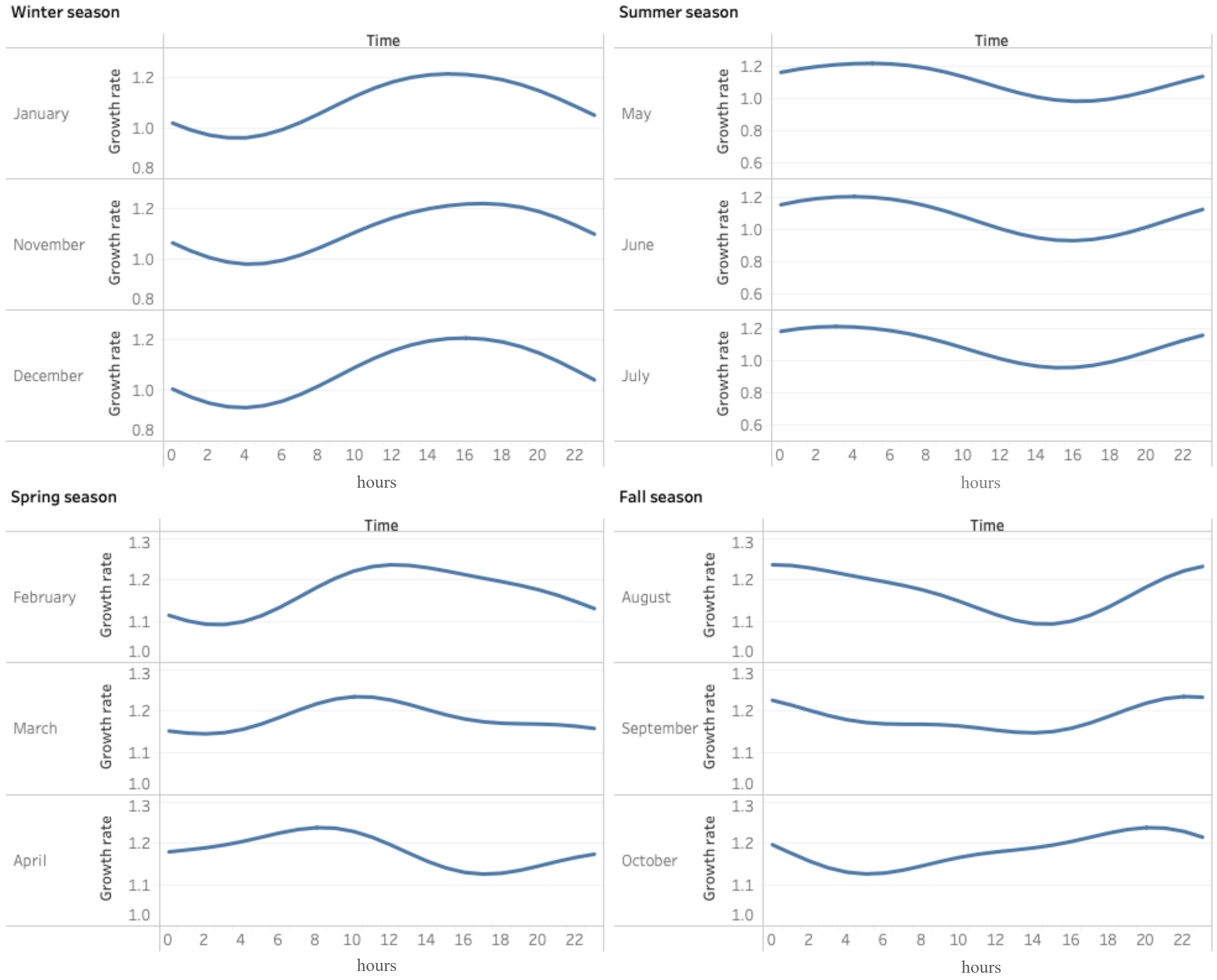

Supplementary Figure 1. **UT vitiation of KHI for different months of the year from Theory**. Diurnal variation of the KHI growth rate similar to Figure 3 (theory panel), but for different months during winter, summer, spring, and fall seasons. Note that the solstices have greater diurnal variation than the equinoxes.

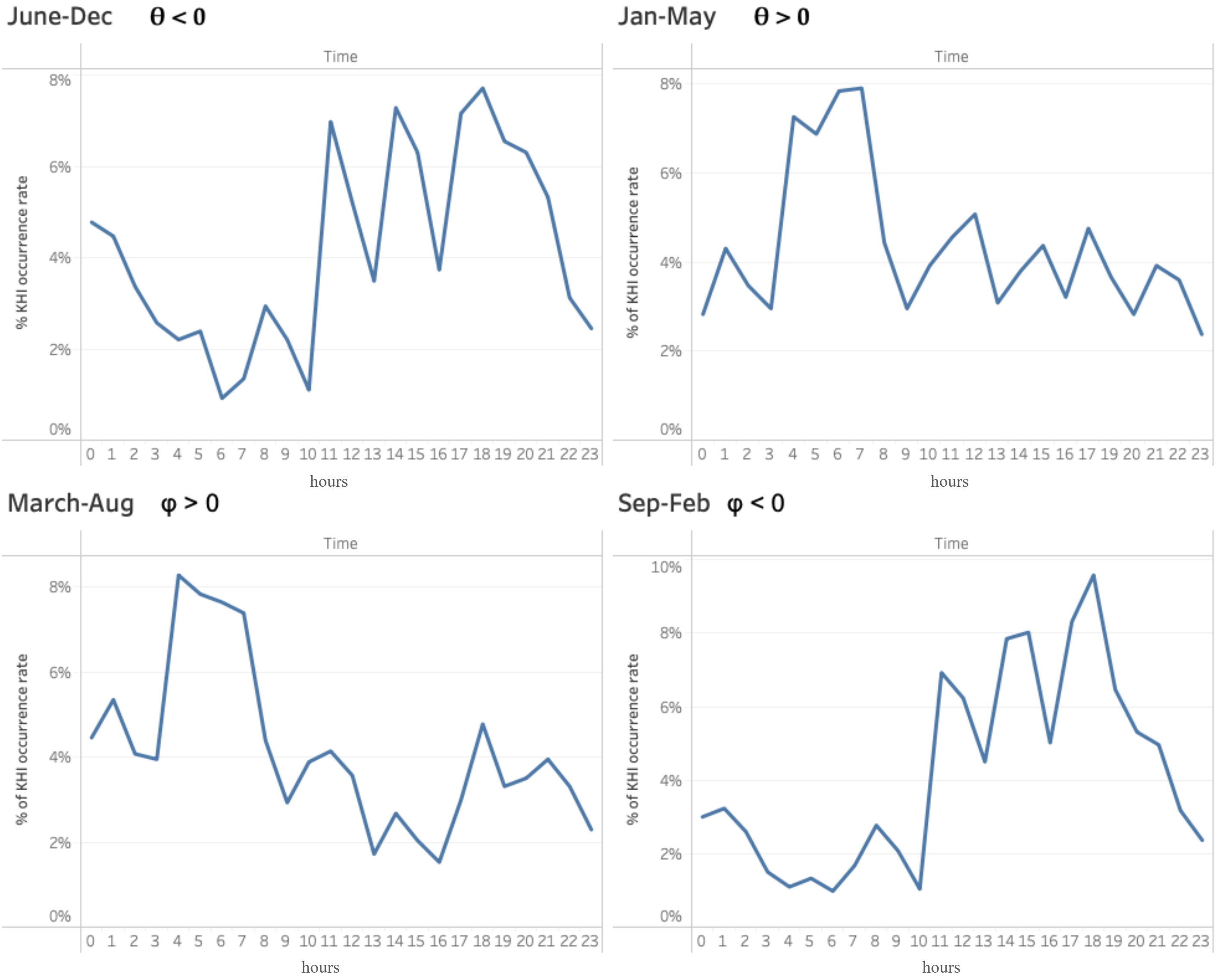

Supplementary Figure 2. **UT vitiation of KHI from observational data**. Top panels show diurnal variations for the months of Jun-December when the angle θ is negative and months of Jan-May when θ is positive. Bottom panels; diurnal variations for months of March-Aug when the angle ϕ is positive and for months Sep-Feb when the angle ϕ is negative.

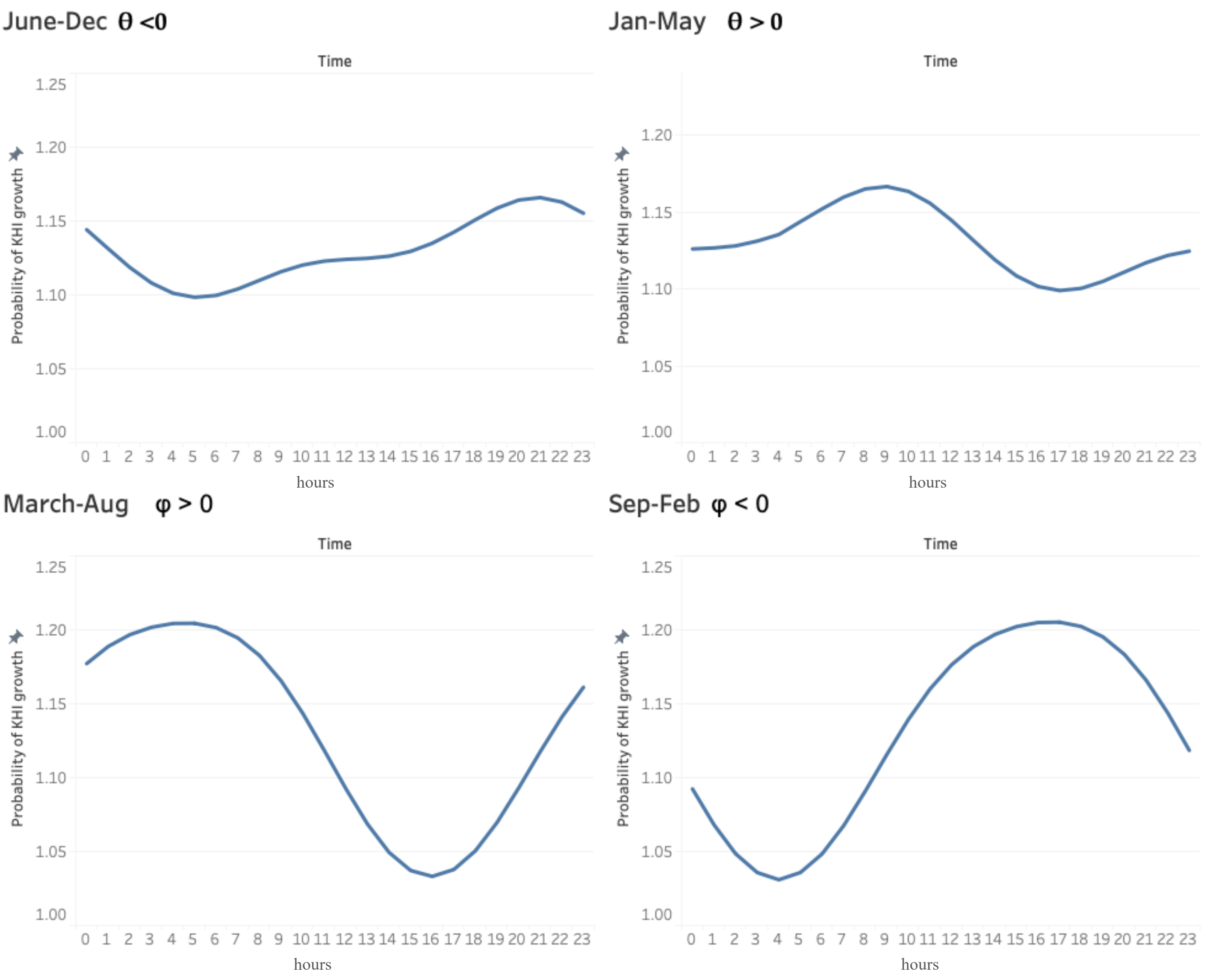

Supplementary Figure 3. **UT vitiation of KHI growth rate from theory**. Similar format as Supplementary Figure 2.,but from theory for comparison with the data.; Top, diurnal variations for the months of Jun-December when the angle θ is negative and months of Jan-May when θ is positive. Bottom, diurnal variations for months of March-Aug when the angle ϕ is positive and for months Sep-Feb when the angle ϕ is negative.

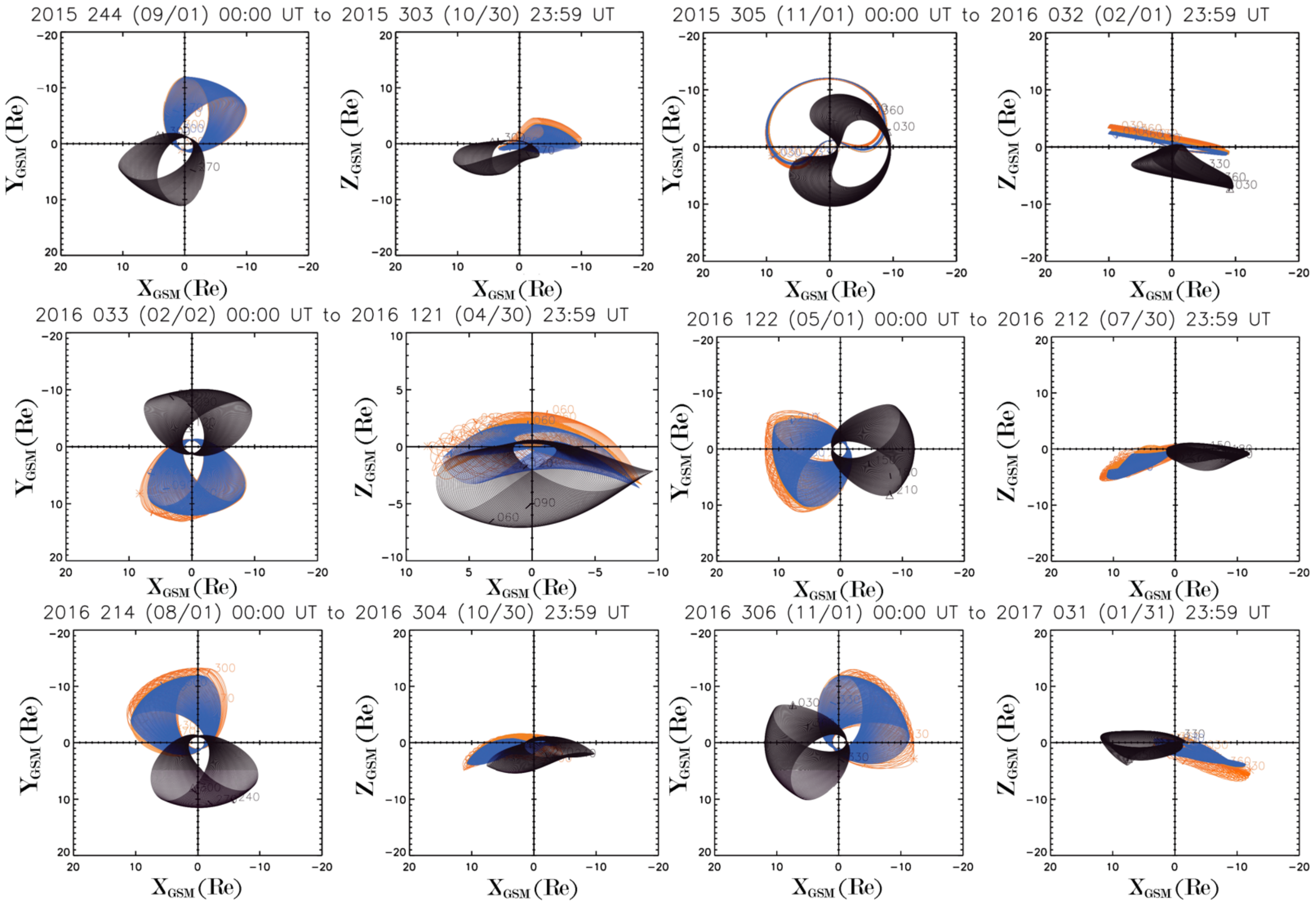

Supplementary Figure 4: The seasonal locations of THEMIS and MMS THEMIS for 2015-2016. THEMIS A, E (depicted by orange lines), THEMIS D (depicted by blue lines), and MMS (depicted by Black lines) orbits in X-Y GSM and X-Z GSM in earth radius (Re) for 2015-2016, showing the possible orbital bias. The figures are from NASA CDAWeb https://sscweb.gsfc.nasa.gov.

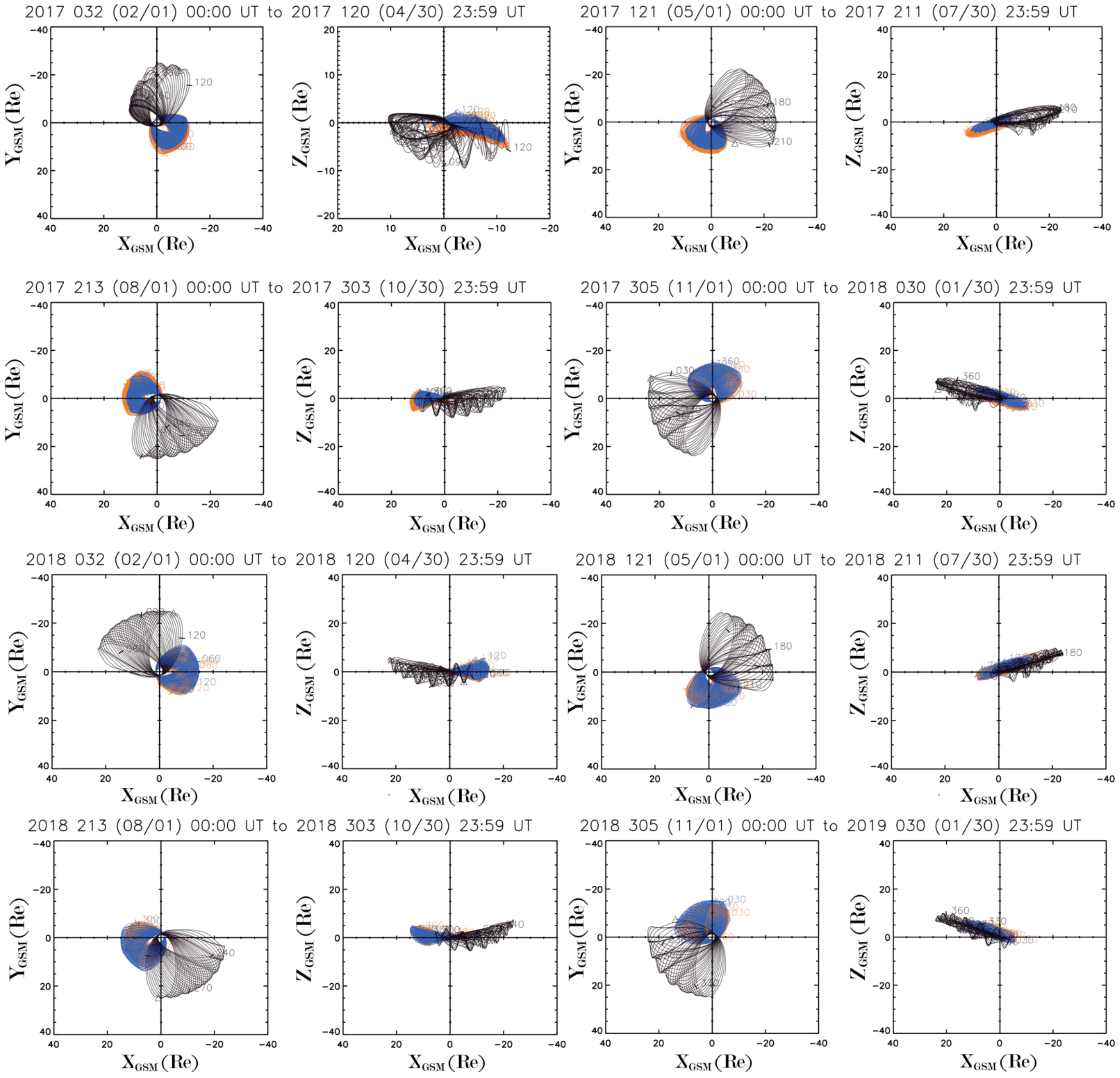

Supplementary Figure 5: The seasonal locations of THEMIS and MMS THEMIS for 2017-2018. THEMIS A, E (depicted by orange lines), THEMIS D (depicted by blue lines), and MMS (depicted by Black lines) orbits in X-Y GSM and X-Z GSM in earth radius (Re) for 2017-2018, showing the possible orbital bias. The figures are from NASA CDAWeb https://sscweb.gsfc.nasa.gov.

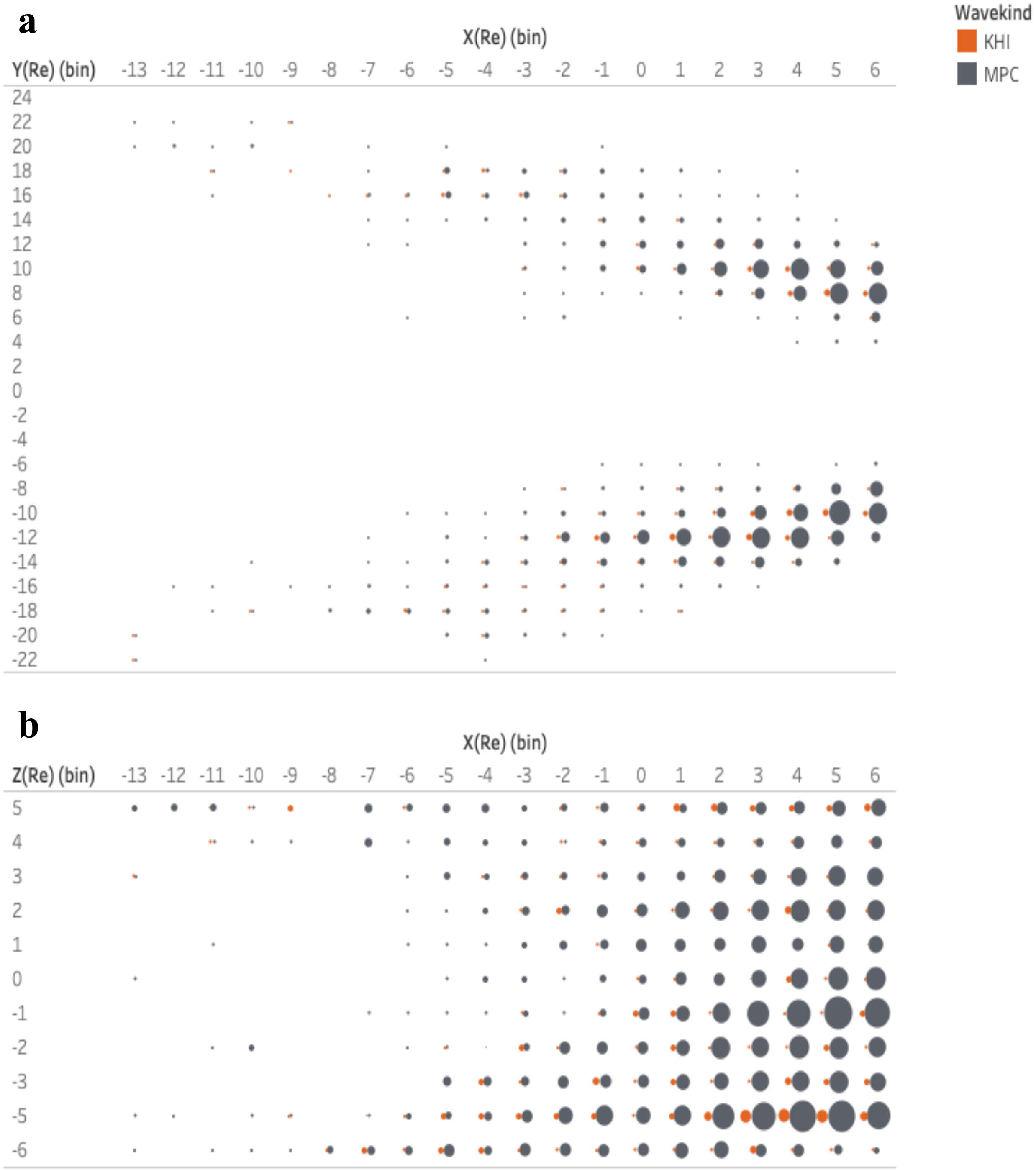

Supplementary Figure 6: Distribution of MPCs/KHWS data in X-Y and X-Z GSM planes. a) in X-Y GSM plane. b) X-Z GSM plane. Orange circles show the relative Kelvin-Helmholtz (KH) wave occurrence rate with respect to the number of boundary crossings, and the grey circles indicate the corresponding number of 5-min boundary crossing intervals in that bin. A larger circle shows a larger number of 5-minute samples.

Supplementary Methods 1: PREDICTION OF KELVIN-HELMHOLTZ HYPOTHESIS:

KHI criteria can be applied to the MP assuming an angle $\boldsymbol{\psi}$ between the magnetic field direction and the velocity shear (Boller and Stolov,1970)[1].

$$(V_I - V_m)^2 > \frac{\rho I+\rho m}{4\pi\rho I\rho m}(B_I\cos\psi_I)^2 + (B_M\cos\psi_m)^2 \qquad \textbf{(1)}$$

The times during the day when the maximums and minimums probabilities of the instability occur is seasonally dependent and are calculated from the instability inequality as in table 1.

Constant refers to Constant: $(\frac{4\pi\rho I\rho m\,(V_I - V_m)2}{\rho I+\rho m})$

Yearly Y and UT variations of the Angles are[2]:

ϕ_{year}=23.4*COS((DOY-172) * (2π/365.25)) (2)

ϕ_{day} =11.2*COS((UT-16.72) *2π/24) (3)

$\phi_{tilt} = \phi_{year} + \phi_{day}$ (4)

θ_{year}=23.4*SIN((172-DOY) * (2π/365.25)) (5)

θ_{day} =11.2*SIN((UT-16.72) *2π/24) (6)

$\theta_{tot} = \theta_{year} + \theta_{day}$ (7)

The IMF "clock angle" in the GSM frame, CA = arctan (|[BY]GSM|/([BZ]GSM).

$CA_{GSM} = CA_{GSE} - \theta_{tot}$ (8)

To calculate the inequality as a function of **the day** and UT, we substitute the angles ϕ_{tot} , and θ_{tot} into the inequality and compute the inequality in GSM coordinate: $(V_I - V_m)^2 > \frac{pI+pm}{4\pi pIpm}[B_I\cos(90-(\text{CA GSM}))^2 + (B_M\cos(90-\phi)^2]$.

$$(V_I - V_m)^2 > \frac{pI+pm}{4\pi pIpm}[(B_I\sin(CAgse - \theta)^2 + (B_M\sin(\phi)^2] \qquad \text{Constant}= \frac{4\pi pIpm\,(V_I - V_m)2}{pI+pm} \qquad (9)$$

$$[(\text{Constant}) - (B_I\sin(CA - \theta)^2 + B_M\sin(\phi))^2] > 0 \qquad (10)$$

For CA=0. $[(\text{Constant}) - (B_I\sin(\theta)^2 + B_M\sin(\phi))^2] > 0$ (11)

For CA=90. $[(\text{Constant}) - (B_I\cos(\theta)^2 + B_M\sin(\phi))^2] > 0$ (12)

Time	Spring Equinox	Fall Equinox	Summer solstice	Winter solstice
04:30 UT	ϕ=-11.5 θ =+23<br>$\psi_{m=}$ 101.5 ψ_I=67<br>[Constant- [(0.16B_I^2) +(0.04B_M^2)]]	ϕ=-11.5 θ=-23<br>$\psi_{m=}$ 101.5 ψ_I =113<br>[Constant-[(0.16B_I^2) + (0.04B_M^2)]	ϕ=+11.5 θ =0<br>$\psi_{M=}$ 101.5 ψ_I =90<br>[Constant-(0.04B_M^2)]<br>**=[Constant -[0.16 B_I^2]]**	ϕ=-35 θ =0<br>$\psi_{M=}$ 125 ψ_I =90<br>[Constant –(0.33B_M^2)]
10:30 UT	ϕ=0 θ =+11.5<br>$\psi_{m=}$ 90. ψ_I=78.5<br>**[Constant –[0.04B_I^2]]**	ϕ=0 θ=-35<br>$\psi_{M=}$ 90. ψ_I =125<br>[Constant-[0.33B_I^2]]	ϕ=+23 θ =-11.5<br>$\psi_{m=}$ 67 ψ_I=101.5<br>[Constant-[(0.01B_I^2) + (0.16B_M^2)]]	ϕ=-23 θ =-11.5<br>$\psi_{m=}$ 113 ψ_I=101.5 P<br>[Constant- [(0.01B_I^2) + (0.16B_M^2)]]
16:30UT	ϕ=+11.5 θ=+23 $\psi_{M=}$ 78.5 ψ_I =67.<br>[Constant-[(0.16B_I^2) + (0.04B_M^2)]]	ϕ=+11.5 θ=-23<br>$\psi_{M=}$ 78.5 ψ_I =113<br>[Constant-[(0.16B_I^2) +(0.04B_M^2)]]	ϕ=35.0 θ=0<br>$\psi_{M=}$ 55 ψ_I =90<br>[Constant – [(0.33B_M^2)]	ϕ=-11.5 θ =0<br>$\psi_{M=}$ 101.5 ψ_I =90<br>[Constant – [(0.04B_M^2)]]<br>**=[Constant -[0.16 B_I^2]]**
22:30 UT	ϕ=0 θ =+35<br>$\psi_{m=}$ 90. ψ_I=55<br>[Constant- [(0.33B_I^2)]]	ϕ=0 θ =-11.5<br>$\psi_{m=}$ 90. ψ_I=101.5<br>**[Constant- [(0.04B_I^2)]]**	ϕ=+23 θ=+11.5. $\psi_{M=}$67 ψ_I=78.5<br>[Constant-[(0.01B_I^2) +(0.16B_M^2)]	ϕ=-23 θ =+11.5<br>$\psi_{m=}$ 113 ψ_I=78.5<br>[Constant-[(0.01B_I^2) + (0.16B_M^2)]]

Supplementary Table 1: Diurnal Variation at solstices and equinoxes. the bold text is the times with minimum magnetic tension, so maximum KH occurrence. The BI/BM is 1/2.

Supplementary Reference:

1. Nowada M, Shue J-H, Russell CT. Effects of dipole tilt angle on geomagnetic activity. Planet Space Sci 57(11): 1254–1259 (2009).
2. Boller BR, Stolov HL. Kelvin-Helmholtz instability and the seasonal variation of geomagnetic activity. J Geophys Res 75 (31): 6073–6084 (1970).
