# Supplementary material for: Seasonal and diurnal variations of Kelvin-Helmholtz Instability at terrestrial magnetopause
Source: Nat Commun. 2023 May 4;14:2513. doi: 10.1038/s41467-023-37485-x (PMC10160038; doi:10.1038/s41467-023-37485-x)
Supplement: Supplementary file 2 — Description of Additional Supplementary Files [file 41467_2023_37485_MOESM2_ESM.pdf]

File Name: Supplementary Data 1

Description: It is the list of Kelvin-Helmholtz Waves Events Using THEMIS and MMS data 2007-2018.

File Name: Source Data File 1

Description: Sheet 1 is the source data For Figure 2. Sheet 2 is the source data for Figure 3, the observational panels, and sheet 3 is the source data for Figure 3, the theory panels.

File Name: Supplementary Code 1

Description: It calculated the magnitude of the stabilizing term, $[-(B^2_I\sin^2(\theta)+B^2_M\sin^2(\varphi))]$.

The Earth's spin axis is inclined by an angle alpha=23.5 and rotates once per year with a period of 365 days, 6 hours, and 9 minutes. The magnetic pole is inclined at an angle φ= 11.2 with respect to the spin axis and rotates about the spin axis once every 23.9344696 hours (a siderial day). We determined the magnetic field direction **for** one year starting with a specified date. For this program, we use January 1, 2020, at 12:00:00 UT. The time is incremented **in** hours from that time. The function JD2GMST returns GMST **in** degrees given Julian's date. We evaluated this function at noon and then interpolated it to get the angle of Earth's rotation axis at each hour relative to the sun-Earth line.
